# Influence of Physical Activity during Pregnancy on Birth Weight: Systematic Review and Meta-Analysis of Randomized Controlled Trials

**DOI:** 10.3390/jcm12165421

**Published:** 2023-08-21

**Authors:** Dingfeng Zhang, Taniya S. Nagpal, Cristina Silva-José, Miguel Sánchez-Polán, Javier Gil-Ares, Rubén Barakat

**Affiliations:** 1 AFIPE Research Group, Faculty of Physical Activity and Sport Sciences-INEF, Universidad Politécnica de Madrid, 28040 Madrid, Spain; zhangdingfeng123@gmail.com (D.Z.); crisverin_29@hotmail.com (C.S.-J.); miguelsanpol@gmail.com (M.S.-P.); barakatruben@gmail.com (R.B.); 2 Faculty of Kinesiology Sport and Recreation, University of Alberta, Edmonton, AB T6G 2R3, Canada; tnagpal@ualberta.ca

**Keywords:** birth weight, macrosomia, low birth weight, gestational age, pregnancy, physical activity

## Abstract

Birth weight is a marker that is often referred to determine newborn health, potential growth trajectories and risk of future disease. Accordingly, interventions to promote appropriate and healthy birth weight have been extensively studied and implemented in pregnancy. In particular, physical activity in pregnancy is recommended to promote appropriate fetal development and newborn birth weight. This systematic review and meta-analyses aimed to summarize the effect of physical activity during pregnancy specifically from randomized controlled trials on the following outcomes: birth weight, macrosomia, low birth weight, being large for the gestational age, and being small for the gestational age (Registration No.: CRD42022370729). 63 studies (16,524 pregnant women) were included. There was a significant negative relationship between physical activity during pregnancy and macrosomia (z = 2.16; *p* = 0.03; RR = 0.79, 95% CI = 0.63, 0.98, I^2^ = 29%, *P_heterogeneity_* = 0.09). No other significant relationships were found. Promoting physical activity during pregnancy may be an opportune time to reduce the risk of future chronic disease, such as obesity, through the prevention of macrosomia and the promotion of appropriate birth weights.

## 1. Introduction

Birth weight is an important and accessible factor to evaluate newborn health and predict growth trajectories and downstream risk of potential chronic disease such as obesity [1]. The World Health Organization defines low birth weight as the weight of a newborn below 2500 g, typically representing the 10th percentile for its gestational age [2]. Newborns that are small for their gestational age (less than the 10th percentile based on gestational age) are at greater risk of having a low birth weight, and associations have also been found with long-term cognitive deficits in childhood and adolescence [3,4]. On the opposite end of the weight spectrum, macrosomia is defined arbitrarily as a birth weight exceeding 4000 g [5]. Babies born with macrosomia have a higher likelihood of encountering adverse delivery outcomes, including shoulder dystocia, brachial plexus injury, clavicular fracture, birth asphyxia, and neonatal mortality [6,7,8]. Moreover, macrosomia increases the risk of undergoing cesarean section and experiencing vaginal and perineal trauma, as well as postpartum hemorrhage [9,10]. Similar complications are associated with newborns that are large for their gestational age (within the 90th percentile based on their gestational age), including prolonged delivery and the risk of injury at birth. Given the risk of complications at either end of the birth weight spectrum, interventions to promote appropriate birth weight are integral.

Physical activity during pregnancy has been described as a modifiable and accessible health behavior to facilitate maternal and newborn health, including the promotion of an appropriate birth weight [11]. International guidelines for prenatal physical activity suggest that all pregnant individuals without contraindications for being active should aim to accumulate 150 min of moderate-intensity activity per week [12]. Contrary to popular social beliefs that physical activity in pregnancy could result in reduced fetal growth and development, no associations have been found with such factors [13]. In fact, being active throughout pregnancy has been shown to advance placenta blood perfusion [14], which can improve nutrient transport and overall placental function and therefore facilitate fetal development [15]. Furthermore, previous reviews have consistently advised that physical activity in pregnancy does not have adverse effects on fetal development [14,16,17].

However, associations between physical activity in pregnancy and birth weight have been inconclusive. For example, a recent systematic review of 32 studies on prenatal physical activity concluded that there is no association between engagement in physical activity throughout pregnancy and newborn body composition markers [18]. Another study that included objective measures of prenatal physical activity via accelerometry suggested that physical activity was correlated with birth weight, and specifically, that aerobic activity reduced birth weight and increased the number of newborns weighing within the appropriate range [19]. Contrarily, a recent meta-analysis of randomized controlled trials that included studies that had assessed gestational weight gain found no associations between prenatal physical activity and large- or small-for-gestational-age newborns [20]. Despite the inconsistencies in the literature regarding birth weight, prenatal physical activity is still recommended, given the several health benefits for both the pregnant person and future child [16]. In fact, a recent expert review emphasized that physical activity in pregnancy should be integrated into standard care, especially given the high quality and strong evidence base supporting its contribution to reduced perinatal complications and improved labor and delivery outcomes [16].

Rather than negating or minimizing the potential benefits of prenatal physical activity on birth weight due to inconsistent results, they indicate that it may be necessary to further examine this relationship via high-quality randomized controlled trials that specifically target these outcomes. Accordingly, the purpose of this systematic review and meta-analysis was to assess the effect of prenatal physical activity on markers of birth weight (i.e., total birth weight and incidences of being large for the gestational age, being small for the gestational age, low birth weight, and macrosomia) from randomized controlled trials. We hypothesized that the amalgamated findings from randomized controlled trials would be in favor of physical activity in pregnancy for reduced risk of being large for the gestational age, being small for the gestational age, having a low birth weight, and macrosomia, along with showing a significant association with reduced total birth weight.

## 2. Methods

A systematic review was developed in accordance with the Preferred Reporting Items for Systematic Reviews and Meta-Analyses (PRISMA) [21]. The protocol was registered in the International Prospective Registry of Systematic Reviews (PROSPERO), Registration No. CRD42022370729.

### 2.1. Eligibility Criteria

The eligibility criteria for this systematic review and meta-analysis was guided by the PICOS framework: participants, interventions, comparisons, outcomes, and study design [20]. The participants included pregnant women, the intervention was physical activity, the comparison was no physical activity/control, and the outcomes were birth weight, being small for the gestational age, being large for the gestational age, and macrosomia.

### 2.2. Population

The population of interest was pregnant individuals without contraindication to exercise or physical activity (following the most recent international clinical guideline about physical activity during pregnancy) [22,23]. Absolute contraindications were characterized by conditions such as ruptured membranes, premature labor, persistent second- or third-trimester bleeding, and other similar factors. On the other hand, relative contraindications were characterized by a history of spontaneous abortion, mild/moderate cardiovascular or respiratory disease, etc. [22,23].

### 2.3. Intervention (Exposure)

We conducted a search to identify physical activity interventions during pregnancy that involved quantifiable forms of physical activity. The focus was on extracting information regarding the program’s reporting of duration, intensity, type of activities, weekly frequency, session duration, participant adherence, and whether supervision was provided.

### 2.4. Comparison

The comparator was no exercise or physical activity (i.e., the control group of the selected studies), normally involving pregnant participants who followed a regular obstetrical follow-up in their health centers.

## 3. Outcome

The main outcomes of the study were birth weight, macrosomia, and low birth weight. Secondary outcomes were being large for the gestational age and being small for the gestational age.

## 4. Data Sources

A comprehensive search was carried out through the Universidad Politécnica de Madrid software in the following databases: Academic Search Premier, ERIC, MEDLINE, SPORTDiscus, OpenDissertations, Clinicaltrials.gov, Web of Science, Scopus, the Cochrane Database of Systematic Reviews, and the Physiotherapy Evidence Database (PEDro). To ensure equality in the selection process, the same article selection criteria were used for all databases, considering differences in controlled vocabulary and rules of selection syntax. The search terms used were:

- English: (physical activity OR exercise OR training OR physical exercise OR fitness OR (strength training) OR physical intervention OR Pilates OR Yoga OR strengthening OR aerobic OR resistance training OR pelvic floor muscle training) AND (pregnancy OR maternal OR antenatal OR pregnant AND (birth weight OR macrosomia OR low birth weight OR large gestational age OR small gestational age) AND (randomized clinical trial OR randomized controlled trial OR RCT).
- Spanish: (actividad física O ejercicio O entrenamiento O ejercicio físico O fitness O entrenamiento de fuerza O intervención de actividad física O Pilates O Yoga O fortalecimiento O aeróbico O entrenamiento de resistencia O fortalecimiento del suelo pélvico) Y (embarazo O materno O antenatal O embarazada Y peso de nacimiento O macrosomía O bajo peso al nacer O gran edad gestacional O pequeña edad gestacional) Y (ensayo clínico aleatorizado O ensayo controlado aleatorizado O ECA).

## 5. Study Selection and Data Extraction

Only randomized controlled trials (RCTs) were selected. Also, systematic reviews previously published in the same field were searched to compare our results. Articles published between 2010 and 2023 written in English and Spanish were considered for the search. Reference lists of selected studies were retrieved to identify other studies that might have been missed by the electronic keyword search.

To ensure compliance with the inclusion criteria, two reviewers (MS and CS) conducted an independent screening of the titles and abstracts retrieved from the electronic searches. The abstracts that met the initial screening were subjected to further analysis. The full texts were screened independently by two reviewers (JG and RB) to identify outcomes of interest for data extraction.

To identify any potential additional studies that were not captured by the electronic searches, the list of references from selected articles was screened. In cases where a study had multiple publications, the most recent or comprehensive publication was chosen as the primary source. However, relevant data from all the publications were extracted to ensure that no valuable information was overlooked.

**Figure 1:**
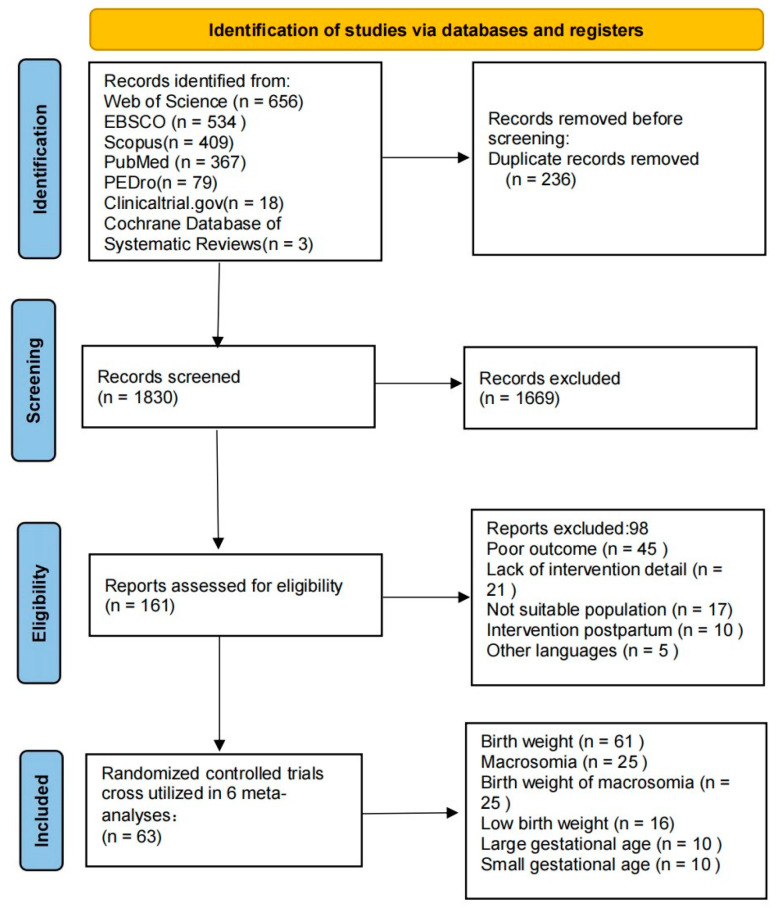
Flow chart of the retrieved and analyzed articles.

For studies where one reviewer (DZ) recommended exclusion, both reviewers (CS and MS) tried to reach a consensus to make a final decision for exclusion or inclusion. In situations of absolute discrepancy, a third reviewer (RB) provided their expert opinion on whether the study should be included or excluded. The study selection process is detailed in Figure 1.

Data extraction tables were created in an Excel sheet. One researcher extracted the data, and then data extraction was independently verified by a content expert to facilitate further analysis. Extracted data were study characteristics (i.e., author last name, year and country), type of article (RCT), total sample size and group sample size, intervention/exposure (exercise prescribed and/or measured), including: frequency, intensity, time and type, supervision of intervention, duration of intervention and adherence of intervention, primary and secondary outcomes (Table 1).

## 6. Quality of Evidence and Risk of Bias Assessments

To evaluate the quality of evidence for each study design and outcome, the Grading of Recommendations Assessment, Development and Evaluation (GRADE) framework was used. This framework provides a standardized and comprehensive approach to assess the strength of the evidence across multiple studies [87].

To evaluate the risk of bias, the Cochrane Handbook was utilized. The potential sources of bias evaluated are selection bias (inadequate randomization procedures for RCTs), performances bias (compliance with the intervention for RCTs), detection bias (flawed outcome measurement), attrition bias (incomplete follow-up and high loss to follow-up), and reporting bias (selective or incomplete outcome reporting) [88].

## 7. Statistical Analysis

Statistical analyses were performed with the software RevMan in its 5.3 version. For continuous variables, birth weight (grams), mean, and standard deviations were recorded. The overall confidence interval (CI) was calculated using the mean difference (MD) [89]. All dichotomous outcomes, macrosomia, low birth weight, being large for the gestational age, and being small for the gestational age were expressed as categorical variables (Yes/No) to calculate the relative risk (RR) [90]. Random effect models were applied. To establish the compensated average in both dichotomous and continuous analyses, a weight system was used that considered the sample size per group, and, generally, these were contributed by each study. To assess the variation in study results between studies (i.e., the degree of heterogeneity), the I^2^ statistic was interpreted using established thresholds: 25% for low heterogeneity, 50% for moderate heterogeneity, and >75% for high heterogeneity [91].

## 8. Results

### 8.1. Study Characteristics

In total, 63 studies that met the inclusion criteria were identified, involving 16,524 pregnant women across 23 countries on five continents. All of the studies were randomized control trials, including 59 exercise interventions only and 4 of exercise and dietary counselling. Studies varied in frequency from 2 to 7 days per week, with low to moderate intensities lasting 15 to 75 min per session. These interventions were carried out during the first, second, and third trimesters, and lasted from 3 to 30 weeks. The types of exercise included walking, stationary cycling, water aerobics, swimming, resistance training, stretching, Pilates, Yoga, pelvic floor muscle training, and combinations of various exercise types. Additional details about the studies can be found in the Table 1. The results of mean birth weight, macrosomia, low birth weights, being large for the gestational age, and being small for the gestational age are presented below.

### 8.2. Risk of Bias Assessment

**Figure 2:**
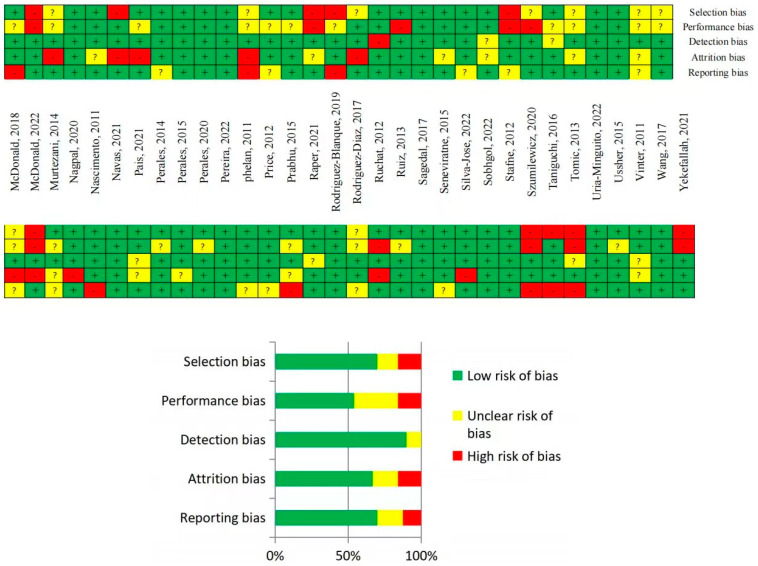
Risk of bias of the included studies [24,25,26,27,28,29,30,31,32,33,34,35,36,37,38,39,40,41,42,43,44,45,46,47,48,49,50,51,52,53,54,55,56,57,58,59,60,61,62,63,64,65,66,67,68,69,70,71,72,73,74,75,76,77,78,79,80,81,82,83,84,85,86].

Collectively, the quality of evidence varied from low to high. In some situations, the blinding of participants to the group (intervention or control group) was not feasible, and it is typically impossible to achieve due to the intervention characteristics (physical activity intervention), resulting in unclear or high risk of bias (performance bias), depending on how it was recorded. Other sources of bias in some cases were the impossibility of finding the published article protocol (to compare the planned and measured outcomes), but also a lack of reporting (or having an uncertain definition) of the randomization process. Overall, the majority of the studies presented a low risk of bias within the five types of bias assessed. The risk of bias analysis is reported in Figure 2.

### 8.3. Effect of Physical Activity during Pregnancy on Birth weight

**Figure 3:**
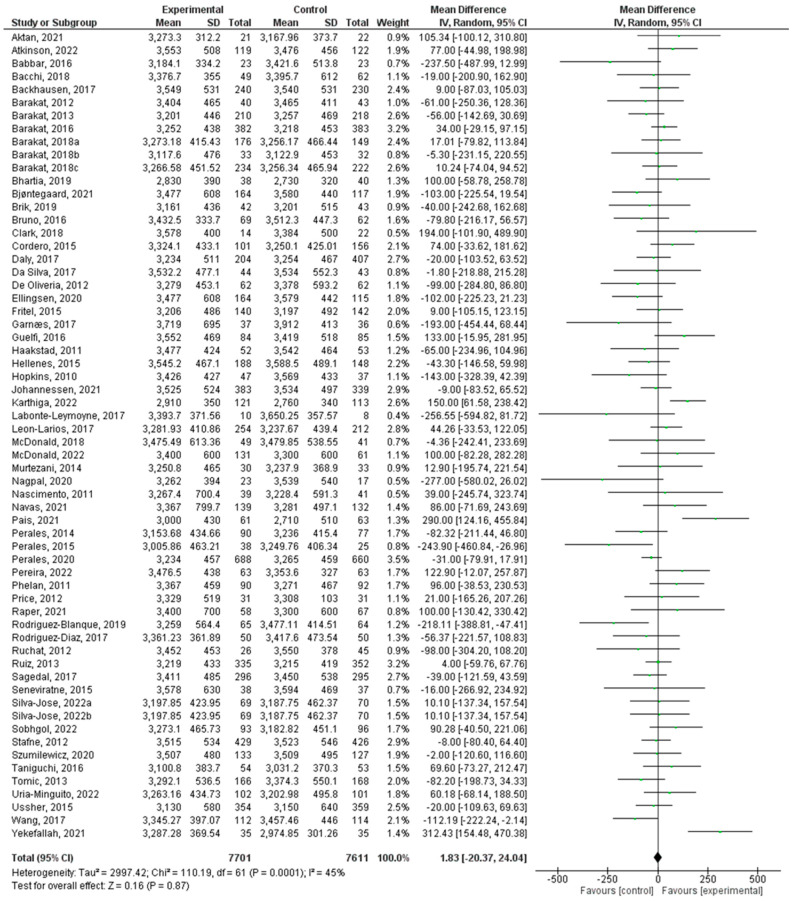
Effect of exercise during pregnancy on birth weight [24,25,26,27,28,29,30,31,32,33,34,35,36,37,38,39,40,41,42,43,44,45,46,47,48,49,50,51,52,53,54,55,56,57,58,59,60,61,62,63,64,65,66,68,69,70,71,72,73,74,75,76,77,78,79,80,81,82,83,84,85,86].

There were a total of 61 studies that were incorporated in this analysis [24,25,26,27,28,29,30,31,32,33,34,35,36,37,38,39,40,41,42,43,44,45,46,47,48,49,50,51,52,53,54,55,56,57,58,59,60,61,62,63,64,65,66,68,69,70,71,72,73,74,75,76,77,78,79,80,81,82,83,85,86]. Regular exercise or physical activity during pregnancy did not have a significant relationship with birth weight (z = 0.11; *p* = 0.91) (Std. Mean Dif., Random, 95% CI = 0.00 (−0.04, 0.05) I^2^ = 43%, *P_heterogeneity_* = 0.0003). The forest plot corresponding to the current meta-analysis is illustrated in Figure 3.

### 8.4. Effect of Physical Activity during Pregnancy on Macrosomia

**Figure 4:**
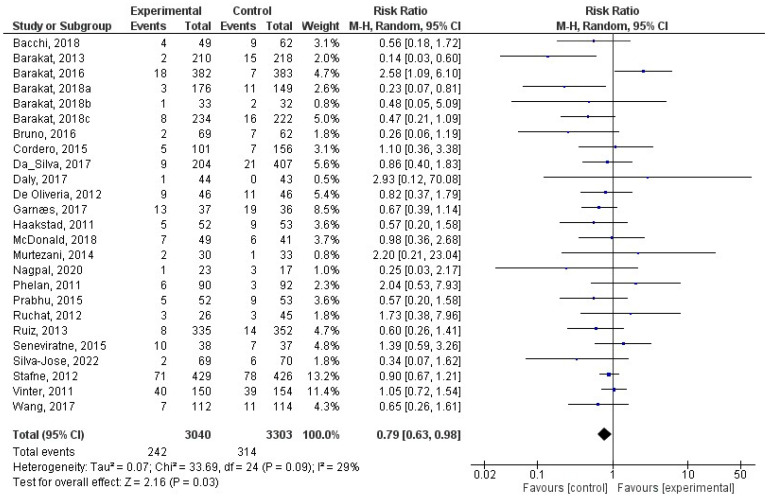
Effect of exercise during pregnancy on macrosomia [27,30,31,32,33,34,38,40,41,42,43,46,48,55,57,58,66,67,72,73,75,76,78,84,85].

**Figure 5:**
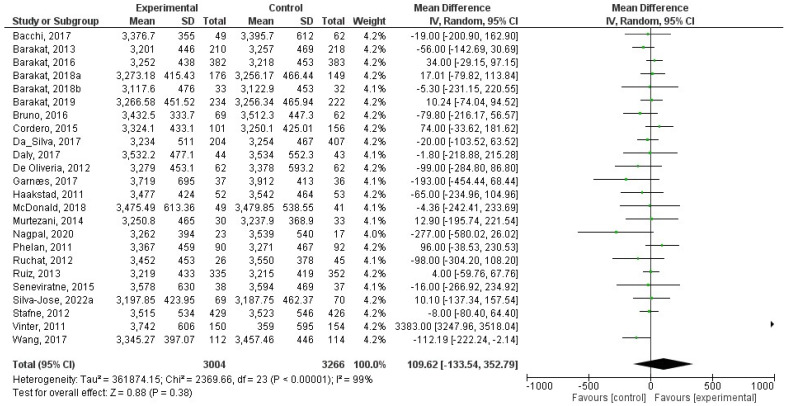
Effect of exercise during pregnancy on birth weight in studies reporting on macrosomia [27,30,31,32,33,38,40,41,42,43,46,48,55,57,58,66,72,73,75,78,84,85].

This analysis included a total of 25 studies [27,30,31,32,33,34,38,40,41,42,43,46,48,55,57,58,66,67,72,73,75,76,78,84,85]. There was a significant relationship (z = 2.16; *p =* 0.03) between physical activity during pregnancy and macrosomia (RR = 0.79, 95% CI = 0.63, 0.98, I^2^ = 29%, *P_heterogeneity_* = 0.09) such that macrosomia occurred less frequently in the exercise group. In Figure 4, the forest plot pertaining to the current meta-analysis is depicted. In addition, we tested only the studies that reported on macrosomia for differences in birth weight, and none were observed (z = 0.86; *p* = 0.39) (Figure 5).

### 8.5. Effect of Physical Activity during Pregnancy on Low Birth Weight

**Figure 6:**
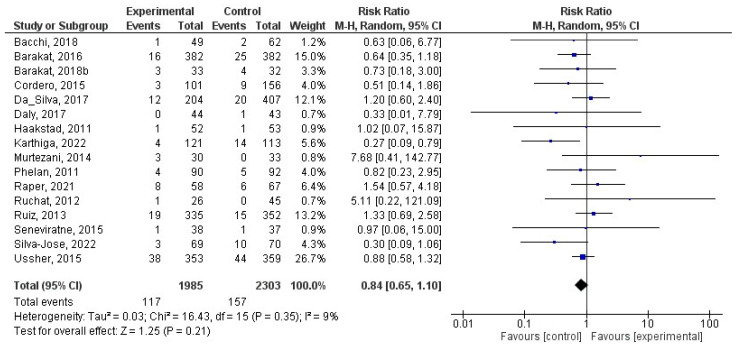
Effect of exercise during pregnancy on low birth weight [27,31,33,40,41,42,48,52,57,66,69,72,73,75,76,83].

This analysis comprised a total of 16 studies [27,31,33,40,41,42,48,52,57,66,69,72,73,75,76,83]. There was no statistically significant association (z = 1.25; *p =* 0.21) between physical activity during pregnancy and the likelihood of low birth weight (RR = 0.84, 95% CI = 0.65, 1.10, I^2^ = 9%, *P_heterogeneity_* = 0.35). Figure 6 displays the forest plot for the present meta-analysis.

### 8.6. Effect of Physical Activity during Pregnancy on Large for Gestational Age

**Figure 7:**
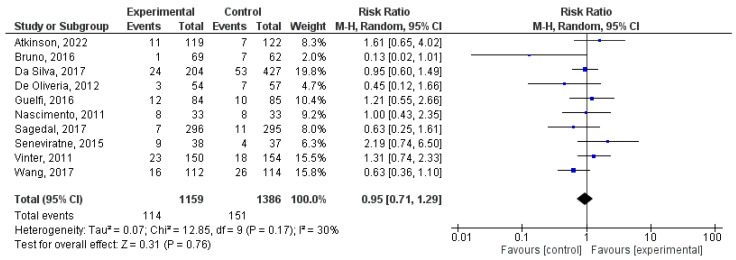
Effect of exercise during pregnancy on being large for gestational age [25,38,41,43,47,59,74,75,84,85].

There was a total of 10 studies that were incorporated into this analysis [25,38,41,43,47,59,74,75,84,85]. There was no difference (z = 0.31; *p =* 0.76) between intervention and control for being large for the gestational age (RR = 0.95, 95% CI = 0.71, 1.29, I^2^ = 30%, *P_heterogeneity_* = 0.17). Figure 7 visually presents the results of the meta-analysis through a forest plot.

### 8.7. Effect of Physical Activity during Pregnancy on Small for Gestational Age

**Figure 8:**
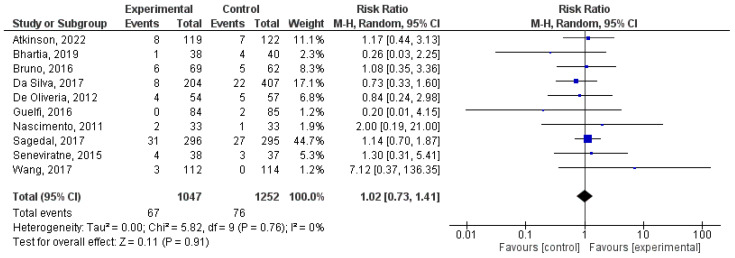
Effect of exercise during pregnancy on being small for gestational age [25,35,38,41,43,47,59,74,75,85].

There was a total of 10 studies that were incorporated into this analysis [25,35,38,41,43,47,59,74,75,85]. Incorporating regular exercise during pregnancy did not cause a significant difference (z = 0.11; *p =* 0.91) for being small for the gestational age (RR = 1.02, 95% CI = 0.73, 1.41, I^2^ = 0%, *P_heterogeneity_* = 0.76). The meta-analysis results are visually presented in Figure 8 through a forest plot.

## 9. Discussion

This systematic review and meta-analysis demonstrated the positive effect of physical activity in pregnancy in reducing the risk of macrosomia by referring specifically to evidence from randomized controlled trials. No other significant associations were found with indices of birth weight, including birth weight as a continuous variable, risk of being small for the gestational age, and having a low birth weight. Birth weight is often used as an accessible marker of newborn health and as an assessment of potential growth trajectories and downstream risk of chronic disease [1,92]. Physical activity during pregnancy may be a key factor in promoting appropriate birth weight, especially contributing to the prevention of macrosomia, and therefore is an important behavior that will facilitate both maternal and child health.

The only significant finding in relation to prenatal physical activity and birth weight was the reduced odds of developing macrosomia. This finding is consistent with previous reviews that have shown reduced odds of macrosomia with physical activity in pregnancy [13,93]. In fact, one systematic review that conducted a sub-analysis of randomized controlled trials only found a 39% reduced risk of macrosomia [13], and our findings further underscored this, thus supporting the effectiveness of prenatal physical activity in the prevention of macrosomia. Macrosomia has shown strong associations with the risk of downstream childhood obesity, and there have been both physiological and environmental mechanisms that have been proposed [94,95]. For example, it is theorized that macrosomia can be a proxy measure for potential adipose tissue function, including overactivity and therefore excess energy storage that can increase the risk for later-life obesity [96]. Moreover, early food restriction practices have been associated with infants who are larger, potentially as an effort to bring their weight into expected trajectories; however, this may be a detriment to appetite regulation [97,98] later on in life. In line with the Developmental Origins of Health and Disease, uterine and early life environments can program downstream childhood obesity, and perhaps prenatal physical activity is a viable factor that can prevent this by reducing the risk of macrosomia [99]. Notably, we also tested if birth weight was different only amongst the studies that reported on macrosomia, and this was not significant. We postulate that this may be due to the fact that macrosomia is defined in absolute values as >4000 g, whereas birth weight is continuous. It may also be due to the heterogeneity amongst included studies in the measurement of weight, as well as the types of exercises performed. Similar findings have been noted about nutrition and exercise interventions in pregnancy, where favorable prevalence outcomes have been found, such as the prevention of excessive gestational weight gain and macrosomia, but no differences were found when weight was measured continuously between intervention and control groups [24,57,70].

This review did not find any significant relationship with prenatal physical activity and birth weight, large- or small-for-gestational-age newborns, and low birth weight. These null findings are consistent with previous systematic reviews, meta-analyses, and observational studies [13,17,93,100]. Previous research has suggested that the relationship between physical activity during pregnancy and birth weight may have an inverted U-shape, such that higher frequencies and intensities of activity may be associated with being small for the gestational age or low birth weight, whereas lower frequencies and intensities of activity could increase risk of being large for the gestational age or macrosomia [101]. Notably, though, engagement in regular moderate levels of physical activity does not increase the risk for small-for-gestational-age and low-birth-weight newborns [13]. Therefore, although our results found no relationship with the lower end of the weight spectrum, it should be highlighted that engagement in physical activity throughout pregnancy does not increase the risk for smaller newborns. A common misconception is that physical activity in pregnancy could be unsafe for fetal development, as energy reserves would be diverted from the placenta, or there is a risk of physical harm [102]. In order to improve knowledge on the safety of maternal physical activity, it is essential that public health messaging should debunk stereotypes or myths that suggest physical activity in pregnancy can deplete or divert energy reserves for fetal growth and development.

Physical activity throughout pregnancy elicits several benefits for maternal and newborn health, including the prevention of perinatal complications such as excessive gestational weight gain and gestational diabetes [103,104]. In the present review, we assessed the direct relationship between engagement in a physical activity intervention and markers of birth weight; however, it should be acknowledged that benefits pertaining to birth weight could be moderated by improvements in other markers of perinatal health. For example, gestational weight gain and birth weight are positively correlated, and therefore, preventing excessive gestational weight gain may also affect the reduction in birth weight [105]. Similarly, gestational diabetes increases the risk for large-for-gestational-age newborns [106]. Physical activity in pregnancy reduces the risk for excessive gestational weight gain by 32% and gestational diabetes by 38% [102,103], and accordingly, may be attributed to also improving newborn birth weight. Physical activity also improves labor and delivery outcomes, including the reduced risk of cesarean section, thus preventing potential complications associated with higher-birth-weight newborns [107]. Taken together, the benefits of physical activity extend beyond indices of weight and affect both the pregnant person and newborn.

Strengths of this review include the inclusion of both English and Spanish articles, expanding the scope of our search in comparison to previous reviews that were restricted to one language, and the inclusion specifically of randomized controlled trials, allowing for the assessment of the features of physical activity interventions that may not be captured through observational studies (e.g., frequency and type of activity). Moreover, randomized controlled trials are deemed to provide more high-quality evidence. However, these results should be interpreted with caution due to the inclusion of studies deemed to be of low quality, as well as the heterogeneity in the contents of the included interventions. Future research should aim to further extrapolate findings based on the intensity of the intervention and types of physical activity. Future comprehensive research should also expand on the inclusion of additional languages.

## 10. Conclusions

By referring to evidence from randomized controlled trials, this review identified that prenatal physical activity could reduce the risk for macrosomia. Prenatal physical activity did not have a significant effect on mean birth weight, small- or large-for-gestational-age newborns, or low birth weight. Importantly, though, prenatal physical activity does not also increase the risk for smaller newborns, and this is an important message that should be widely disseminated to debunk myths associated with reduced or diverted energy reserves for fetal growth and development with active pregnancies. Further research is needed to examine the effect of prenatal physical activity on birth weight, including specific recommended intensity and types of activity, and whether birth weight is moderated through the prevention of other perinatal complications by physical activity, such as excessive gestational weight gain and gestational diabetes.

## Figures and Tables

**Table 1:** Characteristics of the studies analyzed.

Author	Year	Country	Type	N	EG	CG	Intervention Physical Exercise Program							Main Variables Analyzed	Secondary Variables Analyzed
							Freq	Intensity	Duration of Program	Type of Exercise	Superv. Class	Duration of Class	Adh.		
Aktan [24]	2021	Turkey	RCT	43	21	22	2	Mod	8 w	Clinical Pilates exercise	Yes	60 min	-	General anxiety, gestational weight gain	Type of delivery, birth weight
Atkinson [25]	2022	Canada	RTC	241	119	122	3–4	Mod	22 w	Walking	No	25–40 min	80%	Gestational weight gain	Depression, type of delivery, and birth weight
Babbar [26]	2016	USA	RCT	46	23	23	3	Mod	8 w	Yoga	Yes	60 min	80%	Umbilical artery, type of delivery, birth weight	Gestational weight gain
Bacchi [27]	2018	Argentina	RTC	111	49	62	3	Low–Mod	28 w	Aquatic activities	Yes	55–60 min	80%	Gestational weight gain and birth weight	-
Backhausen [28]	2017	Denmark	RCT	516	258	258	2	Low	12 w	Water exercise	No	70 min	76%	Low back pain, birth weight	Type of delivery
Barakat [29]	2012	Spain	RCT	290	138	152	3	Mod	28 w	Aerobic exercise	Yes	40–45 min	-	Type of delivery	Gestational weight gain and birth weight
Barakat [30]	2013	Spain	RCT	510	255	255	3	Mod	28 w	Aerobic, strength, and flexibility exercise	Yes	50–55 min	95%	Gestational diabetes	Gestational weight gain and birth weight
Barakat [31]	2016	Spain	RCT	765	382	383	3	Mod	28 w	Aerobic, strength, and flexibility exercise	Yes	50–55 min	80%	Hypertension	Type of delivery, gestational weight gain, birth weight
Barakat [32]	2018a	Spain	RCT	429	227	202	3	Mod	28 w	Aerobic exercise	Yes	55–60 min	80%	Duration of labor	Type of delivery, use of epidural, birth weight
Barakat [33]	2018b	Spain	RCT	65	33	32	3	Mod	28 w	Aerobic, pelvic floor strength, and flexibility exercise	Yes	55–60 min	85%	Placenta weight	Gestational age, type of delivery, birth weight
Barakat [34]	2018c	Spain	RCT	456	234	222	3	Mod	28 w	Aerobic exercise	Yes	50–55 min	80%	Gestational weight gain	Gestational age, type of delivery, birth weight
Bhartia [35]	2019	India	RCT	78	38	40	1	Mod	12 w	Yoga	Yes	50 min	-	Maternal stress, type of delivery, birth weight	-
							2				No				
Bjøntegaard [36]	2021	Norway	RCT	281	164	117	1	Mod	12 w	Aerobic, strength, and balance exercise	Yes	60 min	-	Type of delivery, birth weight	Physical activity of children at age of seven
							2				No	45 min			
Brik [37]	2019	Spain	RTC	85	42	43	3	55–60% Max HR	29 w	Aerobic, strength, coordination and balance, and pelvic floor exercise	Yes	60 min	70%	Gestational weight gain, fetal cardiac function	Type of delivery, birth weight, gestational age
Bruno [38]	2016	Italy	RTC	131	69	62	3	Mod	16 w	Walking, dietary counselling	No	30 min	-	Gestational diabetes	Gestational weight gain, type of delivery, birth weight
Clark [39]	2018	USA	RTC	36	14	22	3	Mod	20 w	Aerobic	Yes	60 min	-	Gestational weight gain	Type of delivery, birth weight
Cordero [40]	2015	Spain	RCT	257	101	156	2	50–55% Max HR	26 w	Aerobics in gym hall	Yes	50–60 min	80%	Gestational diabetes	Gestational weight gain, type of delivery, birth weight
							1			Aquatic activity					
Da Silva [41]	2017	Brazil	RTC	639	213	426	3	Mod	16 w	Aerobic, strength training	Yes	60 min	70%	Preterm birth and pre-eclampsia	Gestational weight gain, birth weight
Daly [42]	2017	Ireland	RCT	88	44	44	3	Mod	26 w	Aerobic, pelvic floor exercise	Yes	50–60 min	80%	Maternal fasting plasma glucose	Type of delivery and birth weight
De Oliveria [43]	2012	Brazil	RTC	111	54	57	3	60–80% Max HR	25 w	Walking	Yes	15–40 min	85%	VO2max, birth weight and gestational age	-
Ellingsen [44]	2020	Norway	RTC	279	164	115	1	Mod	12 w	Aerobic activity and strength exercise	Yes	60 min	-	Neurodevelopment in 7-year-old children	Gestational age, birth weight, type of delivery
							2				No	45 min			
Fritel [45]	2015	France	RCT	282	140	142	1	Low	8 w	Pelvic floor training	Yes	20–30 min	-	Urinary incontinence	Type of delivery and birth weight
Garnaes [46]	2017	Norway	RCT	74	38	36	3	Mod	20 w	Aerobic, strength training	Yes	60 min	-	Birth weight	Type of delivery, perineal tears, gestational age
							2			-	No	50 min			
							7			Pelvic floor training	No	1 min			
Guelfi [47]	2016	Australia	RCT	172	85	87	3	Mod	14 w	Home-based stationary cycling program	Yes	20–60 min	-	Gestational diabetes	Type of delivery, birth weight
Haakstad [48]	2011	Norway	RCT	105	52	53	2	Mod	12 w	Aerobic dance and strength training	Yes	60 min	80%	Birth weight	Gestational age, type of delivery
							1				No	30 min			
Hellenes [49]	2015	Norway	RCT	336	188	148	1	Mod	16 w	Aerobic activity	Yes	30 + min	-	Cognitive, language and motor domains of children	Gestational age, birth weight, and type of delivery
							2				No				
Hopkins [50]	2010	New Zealand	RCT	84	47	37	5	65% VO2max	16 w	Stationary cycling program	No	40 min	-	Birth weight, gestational age	-
Johannessen [51]	2021	Norway	RCT	722	383	339	1	Mod	12 w	Aerobic, strength and pelvic floor exercise	Yes	55–70 min	-	Urinary incontinence at 3 months postpartum	Type of delivery, episiotomy, epidural, duration of labor, birth weight
							2				No	45 min			
Karthiga [52]	2022	India	RCT	234	121	113	7	Mod	20 w	Yoga 5 sessions of Yoga techniques	No	60 min	-	Gestational hypertension	Type of delivery, duration of labor, birth weight
Labonte-Leymoyne [53]	2017	Canada	RCT	18	10	8	3	55% VO2max	24 w	Aerobic exercise	Yes	20 + min	-	Neuroelectric response of the neonatal brain	Maternal weight gain, birth weight
Leon-Larios [54]	2017	Spain	RCT	466	254	212	5	Low	6 w	Perineal massage and pelvic floor exercise	No	18–23 min	-	Perineal tear and episiotomy	Type of delivery, duration of labor, birth weight, and epidural analgesia
McDonald [55]	2018	USA	RCT	90	49	41	5	55–69% Max HR	20 w	Walking program	No	40 min	-	Preeclampsia and pathophysiological progress of preeclampsia	Gestational weight gain and birth weight
McDonald [56]	2022	USA	RCT	192	131	61	3	Mod	24 w	Aerobic and resistance training	Yes	50 min	80%	Gestational weight gain, type of delivery and birth weight	-
Murtezani [57]	2014	Republic of Kosovo	RCT	63	30	33	3	Mod	20 w	Aerobic and strength exercise	Yes	40–45 min	85%	Birth weight and gestational age	-
Nagpal [58]	2020	Canada	RCT	40	23	17	3	Mild	11 w	Walking program + nutrition	Yes	25–40 min	80.2%	Scoring women on meeting the intervention goals	Gestational weight gain, birth weight, macrosomia, and low birth weight
Nascimento [59]	2011	Brazil	RCT	80	39	41	5	Low–Mod	17 w	Aerobic exercise Walking	No	40 min	62.5%	Gestational weight gain	Birth weight
Navas [60]	2021	Spain	RCT	294	148	146	3	55–65% Max HR	20 w	Aquatic exercise	Yes	45 min	-	Postpartum depression, quality of life, and quality of sleep	Gestational age, birth weight
Pais [61]	2021	India	RCT	124	61	63	7	Low	20 w	Yoga One-to-one Yoga session for 7 days	No	45 min	-	Preeclampsia and gestational diabetes	Gestational age, duration of labor, type of delivery, birth weight
Perales [62]	2014	Spain	RCT	167	90	77	3	55–60% Max HR	29 w	Aerobic activity	Yes	55–60 min	-	Prenatal depression	Gestational weight gain, birth weight, and type of delivery
Perales [63]	2015	Spain	RCT	63	38	25	3	55–60% Max HR	28 w	Aerobic dance, pelvic floor muscle training	Yes	55–60 min	80%	Fetal and maternal heart rate	Gestational weight gain, birth weight, type of delivery
Perales [64]	2020	Spain	RCT	1348	688	660	3	Light–Mod	30 w	Aerobic, pelvic floor exercise	Yes	50–55 min	95%	Gestational weight gain, hypertension, and gestational diabetes	Type of delivery, birth weight, gestational age
Pereira [65]	2022	Portugal	RCT	126	63	63	3	55–69% Max HR	3 w	Walking	Yes	30 min	-	Rate of labor induction	Type of delivery, birth weight
Phelan [66]	2011	USA	RCT	363	179	184	7	Low	26 w	Walking	No	30 min	-	Gestational weight gain	Gestational hypertension, birth weight, and type of delivery
Price [67]	2012	USA	RCT	62	31	31	4	Mod	23 w	Aerobic exercise	Yes	45–60 min	-	Birth weight	Duration of labor, type of delivery
Prabhu [68]	2015	India	RCT	105	52	53	2	Mod	12 w	Aerobic dance	Yes	45 min	80%	Birth weight	-
							1				No	30 min			
Raper [69]	2021	USA	RCT	125	58	67	3	Mod	24 w	Aerobic	Yes	50 min	80%	Gestational diabetes, type of delivery, and birth weight	-
Rodriguez-Blanque [70]	2019	Spain	RTC	129	65	64	3	Mod	17 w	Aquatic physical exercise	Yes	60 min	-	Laceration and episiotomy rates	Type of delivery, birth weight, and anesthesia
Rodriguez-Diaz [71]	2017	Spain	RCT	100	50	50	2	Mod	8 w	Pilates	Yes	40–45 min	90%	Gestational weight gain, blood pressure, strength, flexibility, and spinal curvature	Type of delivery, episiotomy, analgesia, and birth weight
Ruchat [72]	2012	Canada	RCT	71	26	45	1	Mod	22 w	Walking	Yes	25–40 min	-	Gestational weight gain, birth weight	-
							2–3				No				
Ruiz [73]	2013	Spain	RCT	962	481	481	3	Light–Mod	28 w	Aerobic and resistance exercise	Yes	50–55 min	97%	Gestational weight gain	Birth weight, type of delivery
Sagedal [74]	2017	Norway	RCT	591	296	295	2	Mod	24 w	Aerobic, strength training. Dietary counselling	Yes	60 min	-	Gestational weight gain, birth weight	Gestational age, perineal tear
Seneviratne [75]	2015	New Zealand	RCT	75	38	37	3–5	Mod	16 w	Stationary cycling program	No	15–30 min	-	Birth weight, type of delivery	Gestational weight gain, gestational age
Silva-Jose [76]	2022	Spain	RCT	139	69	70	3	55–65% Max HR	30 w	Aerobic exercise	Yes	55–60 min	80%	Gestational weight gain	Birth weight, type of delivery
Sobhgol [77]	2022	Australia	RCT	200	100	100	7	Low	16 w	Pelvic floor muscle exercise	No	30 min	50%	Female sexual function	Type of delivery, perineal tear, episiotomy, duration of labor, and birth weight
Stafne [78]	2012	Norway	RCT	761	396	365	2	Mod–High	12 w	Aerobic, strength, pelvic floor training	Yes	60 min	55%	Urinary and anal incontinence	Type of delivery, birth weight
							1				No	45 min			
Szumilewicz [79]	2020	Poland	RCT	260	133	127	3	Low–Mod	24 w	Aerobic, resistance, pelvic floor muscle training	Yes	60 min	-	Urinary incontinence 2 months and 1 year postpartum	Type of delivery, birth weight, duration of labor, analgesia
Taniguchi [80]	2016	Japan	RCT	118	60	58	3	Mod	6 + w	Walk briskly	Yes	30 min	80%	Type of delivery, birth weight	-
Tomic [81]	2013	Croatia	RCT	334	166	168	3	60–75% Max HR	28 w	Aerobic exercise	Yes	50 min	80%	Macrosomia, birth weight, type of delivery, gestational weight gain	-
Uria-Minguito [82]	2022	Spain	RCT	203	102	101	3	65–70% Max HR	28 w	Aerobic exercise	Yes	50–60 min	-	Gestational diabetes	Gestational weight gain, type of delivery, birth weight
Ussher [83]	2015	UK	RCT	789	394	395	3–4	Low	6 w	Exercise on a treadmill	Yes	20 min	88.8%	Continuous smoking abstinence	Type of delivery, birth weight
Vinter [84]	2011	Denmark	RCT	304	150	154	7	Mod	24 w	Aerobic exercise, dietary counselling	No	30–60 min	-	Gestational weight gain	Birth weight
Wang [85]	2017	China	RCT	226	112	114	3	55–65% Max HR	24 w	Stationary cycling program	Yes	45–60 min	75%	Gestational diabetes	Birth weight, macrosomia
Yekefallah [86]	2021	Iran	RCT	70	35	35	2	Low–Mod	11 w	Yoga	Yes	75 min	-	Episiotomy, perineal tear, type of delivery	Birth weight, gestational age, duration of labor

Author: first author last name; Year: year of study; Country: country where the article was developed (usually in the method part); Type: type of article, if it is a randomized clinical (or controlled) trial, RCT is indicated. N: total number of women analyzed. Those of the GI and those of the CG have to coincide. EG: Number of women analyzed in the intervention group. CG: number of women analyzed in the control group. Freq: weekly frequency of exercise sessions (3 days a week, 2, etc.). Intensity: moderate, high...; Duration of program: program time. If the program lasted 10 weeks, or if it started in week 12 and ended in week 28, it is described as being 16 weeks long. Type of exercise: aerobic, muscle strengthening, etc. Superv. Classes: whether or not there was supervision. Duration of class: minutes of each session. Adh.: adherence of the participants to the intervention (%). This indicates how many women attended. Main variables analyzed: lists all the main variables of the study. This is usually in the method section in “outcomes”, and they appear as “main outcomes”. If main does not appear, they are the first. You will find it in several places. Secondary variables: the same as before, but secondary. In the same study, this may involve different types of exercises, varying durations for each exercise, and both supervised and unsupervised exercises.

## Data Availability

Not applicable.
